# Supplementary material for: Cosmic Whirl: Navigating the Comet Trail in DNA: H2AX Phosphorylation and the Enigma of Uncertain Significance Variants
Source: Genes (Basel). 2024 Jun 1;15(6):724. doi: 10.3390/genes15060724 (PMC11202575; doi:10.3390/genes15060724)
Supplement: Supplementary file 1 [file genes-15-00724-s001.zip › genes-2990258-supplementary.pdf]

Supporting Information

Cosmic Whirl: Navigating the Comet Trail in DNA: H2AX Phosphorylation and the Enigma of Uncertain Significance Variants

Sevdican Ustun Yilmaz[1], Nihat Bugra Agaoglu[2,3], Karin Manto[4], Meltem Muftuoglu[1,5], **Ugur Özbek[1,4,6]**

[1] Department of Medical Biotechnology, Institute of Health Sciences, Acibadem Mehmet Ali Aydinlar University, 34752 Istanbul, Türkiye

[2] Department of Medical Genetics, Umraniye Training and Research Hospital, University of Health Sciences, Istanbul, Turkey, 34764 Istanbul, Türkiye

[3] IKF Institut für Klinische Krebsforschung GmbH, 60488, Frankfurt am Main, Germany

[4] Department of Genome Studies, Institute of Health Sciences, Acibadem Mehmet Ali Aydinlar University, Istanbul, Turkey, 34752 Istanbul, Türkiye

[5] Department of Molecular Biology and Genetics, Faculty of Engineering and Natural Sciences, Acibadem Mehmet Ali Aydinlar University, 34752 Istanbul, Türkiye

[6] Izmir Biomedicine and Genome Center (IBG), 35340 Izmir, Türkiye

*Present address:
Uğur Özbek
Dokuz Eylül University
IBG-Izmir BioMedicine and Genome Center
15 July Health and Art Campus
Mithatpasa St. 58/5 Balcova,
Izmir/Türkiye
Tel: +90 (232) 299 41 00
e-mail: ugur.ozbek@ibg.edu.tr

Materials and Methods

Subjects and selection of missense VUSs in the *BRCA2* gene

We retrospectively analyzed the NGS-based hereditary cancer panel results of 922 index cases or the cases that were tested due to a positive family history, who were referred to the Hereditary Cancer Clinic in the Genomic Laboratory (GLAB) of Umraniye Training and Research Hospital [18] and Medical Genetics Department of Acibadem Maslak Hospital, Istanbul, Türkiye, between November 2017 and June 2022. Clinical Geneticists at both hospitals reviewed patients' medical and family histories before and after genetic testing, using the National Comprehensive Cancer Network (NCCN) version when the patients were seen and

ACMG/AMP guidelines 11]. Genetic testing reports were performed on the *APC, ATM, BARD1, BRCA1, BRCA2, BRIP1, CDH1, CHEK2, EPCAM, FAM175A, MLH1, MRE11, MSH2, MSH6, MUTYH, NBN, PALB2, PMS2, PTEN, RAD50, RAD51C, RAD51D, STK11, TP53* and *XRCC2* genes which are associated with hereditary cancer.

The cases with *BRCA2* variants were searched in the ClinVar database and missense SNV VUSs, conflicting interpretations of pathogenicity (CIP), and not-reported (NR) variants were filtered (Table S1). We collected clinical information (Table S2). We excluded some of them from the study for the following reasons: they were male, lost to follow-up, not HBOC, had variations in other genes, had travel problems, died, or didn't want to participate (Table S2). We collected the detailed history of each patient/participant and their relatives for oncological diseases (Table S3). Besides, participants replied to a questionnaire during their visit to the clinic (Table S4).

Data collection about variants

Selected variants frequencies in the population were collected from gnomAD version v4.0.0 We used dbNSFP database algorithms to consider the alterations in non-synonymous amino acids in the *BRCA2* gene likely to affect gene functioning. Taking into account the results in this database, variants were analyzed with *in-silico* prediction tools to see the variant's pathogenicity on the protein level, such as SIFT, PolyPhen-2, BayesDel (addAF and noAF), and MetaRNN. In addition, we used the Varsome human genomic variant search engine and DeMAG (deciphering mutations in actionable genes) variants effect predictor tool to collect more information about the variants from global experts.

Peripheral blood mononuclear cells (PBMCs) isolation

Venous blood samples were collected into 10-mL EDTA tubes (BD Vacutainer, USA). PBMCs were separated from whole blood according to the manufacturer's instructions with Ficoll-Paque PLUS (Cytiva, USA) density gradient solution. Briefly, the study participants' blood was mixed with an equal volume of phosphate-buffered saline (PBS) buffer (Gibco by Life Technologies, Washington, DC, USA) and then layered slowly on top of the density gradient solution. After centrifugation, PBMCs were collected from the layer between the Ficoll and the plasma of the blood and centrifugated with PBS. Isolated PBMCs were resuspended in advanced RPMI 1640 medium (Gibco) with 10% FBS (Gibco), and then cells were counted.

During the assay optimization experiments, we closely monitored the viability of PBMCs to distinguish between live and dead cells pre-and post-treatment with Dox. The trypan blue exclusion method was employed using the TC20 automated cell counter device (BioRad, USA). Under the study's experimental conditions, the administration of Dox did not significantly impact the viability of PBMCs. Any data from samples with compromised viability was omitted from our measurements.

Single-cell gel electrophoresis (Comet assay)

Comet assays were performed under alkaline conditions using the CometAssay® Kit (Trevigen, USA) according to the manufacturer's instructions and as described in Ogulur et al. (2021). We made some modifications to these protocols. Briefly, $1x10^6$ PBMCs were treated with 0.5 μM doxorubicin (CST, USA) for 1 h at 37˚C. The treated and untreated samples were centrifuged and washed with ice-cold Dulbecco's PBS (Life Technologies, USA). PBMCs mixed with 0.5%

low melting point agarose in a 1:10 ratio and layered onto comet slides. Slides were incubated at 4˚C for 1 h in the dark. Then, slides were transferred to pre-chilled lysis solution at 4˚C overnight in the dark. After the lysis, slides were immersed into a freshly prepared alkaline unwinding solution at 4˚C for 1 h in the dark. Electrophoresis was performed at 20V at 4˚C for 40 min in the dark. The slides were washed twice with dH_2O for 5 min and then with 70% ethanol for 5 min. Drops were drained at 37˚C for 20 min, then stained with SYBR gold solution (Invitrogen, USA). Slides were analyzed, 100 cells were counted using the Vert.A1 fluorescence microscope (Zeiss, Germany) at X10 magnification, and images were captured using the ZEN software. The captured cell images were scored by the CometScore 2.0.0.38 TriTek program (CometScore Comet Scoring Software, 2004. We analyzed the data by considering the percentage of DNA present in the comet's "tail" as mentioned in the review of statistical comet analysis methods -. We compared 100 cells per slide and two slides per person. We excluded hedgehog cells to calculate the DNA percentage ranges in untreated and treated cells. Two single-blind researchers counted the slides.

Western blotting gamma H2A histone family member X (γH2AX) assay

Whole-cell extracts of PBMCs were prepared using radioimmunoprecipitation assay (RIPA) buffer (50 mM Tris-HCL pH:7.4, 150 mM NaCl, 1 mM EDTA, 1 mM NaF, 1 mM Na_3VO_4, 0.5% NP-40, 0.5% Triton X-100, 0.5 mM PMSF, 1 mM DTT, 1X protease/phosphatase inhibitor (Cell Signaling Technology, USA)). Using BSA as a standard, protein concentration was measured with Bradford protein assay (Bio-Rad, USA). Then, proteins were separated using the SDS-PAGE and transferred to polyvinylidene fluoride (PVDF) membranes (Bio-Rad, USA). Membranes blocked in 5% milk powder (Bio-Rad, USA) for 1 h at room temperature. Primary antibody incubation was performed overnight at 4°C with phospho-Histone-H2AX (Ser 139) (CST-9718S) and β-Actin (CST-3700S). Secondary antibody incubation was performed with anti-mouse IgG HRP-linked (CST-7076S) and anti-rabbit IgG HRP-linked (CST-7074S) antibodies for 1 h at room temperature. Membranes imaged using an electrochemiluminescence (BIO-RAD ChemiDoc™ MP Imaging System) system with Pierce™ ECL Plus Western-Blotting Substrate (Thermo Fisher, USA) or SuperSignal™ West Femto Maximum Sensitivity Substrate (Thermo Fisher, USA). Western-blot experiments were performed in triplicate.

Statistical analysis

All graphs were plotted using the GraphPad Prism 9 software. Comet assay data were presented as the means of % tail DNA ± standard deviation of the mean. All graphs were obtained for the samples according to their genetic status: non-carrier (NC), pathogenic variant carrier (PC), or VUS-Cs. Statistical analysis was performed using GraphPad Prism 9, considering the pooled samples. The Anderson-Darling, D'Agostiono, Shapiro-Wilk, and Kolmogorov-Smirnov normality tests were performed for the comet assays and γH2AX expression to examine if subjects followed a Gaussian distribution. Paired t-test was applied to compare subjects before and after 0.5 μM 1 h Dox-induction. Welch's ANOVA test was performed to compare the different groups of subjects. $P<0.05$ indicated a statistically significant difference; results $p\leq 0.05$ were considered statistically significant (GraphPad InStat).

Supporting Information Table S1. **A** list of all BRCA2 variants in the cohort

**Nucleotide**	**Protein**	**Clinical Significance**	**Molecular Consequence**	**Variant Type**
c.10078A>G	p.Lys3360Glu	VUS	SNV	Missense
c.1013del	p.Ala338fs	P	Deletion	Frameshift
c.10240A>G	p.Thr3414Ala	CIP: VUS (3); LB (5)	SNV	Missense
c.1114A>C	p.Asn372His	B	SNV	Missense
c.1414C>T	p.Gln472Ter	P	SNV	nonsense
c.1507A>T	p.Lys503Ter	P	SNV	nonsense
c.1688G>C	p.Trp563Ser	NR	SNV	Missense
c.2240A>G	p.Glu747Gly	CIP: VUS (9); B (1); LB (1)	SNV	Missense
c.2467G>A	p.Ala823Thr	CIP: VUS (1); LB (1)	SNV	Missense
c.2779A>G	p.Met927Val	CIP: VUS (6); LB (5)	SNV	Missense
c.3032C>G	p.Thr1011Arg	CIP: VUS (9); LB (4)	SNV	Missense
c.3199del	p.Thr1067fs	P	Deletion	Frameshift
c.3302A>G	p.His1101Arg	CIP: VUS (6; LB (3)	SNV	Missense

c.3310A>C	p.Thr1104Pro	CIP: VUS (6); LB (1)	SNV	Missense
c.3318C>G	p.Ser1106Arg	CIP: VUS (5); LB (1)	SNV	Missense
c.3451_3454del	p.Thr1150_Ile1151insTer	LP	Deletion	nonsense
c.3503T>A	p.Met1168Lys	CIP: VUS (6); LB (1)	SNV	Missense
c.3516G>A	p.Ser1172=	B	SNV	synonymous
c.3751dup	p.Thr1251fs	P	Duplication	Frameshift
c.3847_3848del	p.Val1283fs	P	Deletion	Frameshift
c.3910A>C	p.Thr1304Pro	NR	SNV	Missense
c.4205A>G	p.Asn1402Ser	CIP: VUS(1), LB(1)	SNV	Missense
c.4277C>T	p.Thr1426Ile	CIP: VUS (5); LB (2)	SNV	Missense
c.4284dup	p.Gln1429fs	P	Duplication	Frameshift
c.4327T>C	p.Phe1443Leu	CIP: VUS (1); LB (1)	SNV	Missense
c.4446_4451dup	p.Glu1482_Thr1483dup	VUS	Duplication	inframe insertion
c.4585G>A	p.Gly1529Arg	B	SNV	Missense
c.4631dup	p.Asn1544fs	P	Duplication	Frameshift
c.4732T>G	p.Leu1578Val	LB	SNV	Missense

c.4957A>G	p.Thr1653Ala	CIP: VUS (7); LB (2)	SNV	Missense
c.5125G>T	p.Asp1709Tyr	CIP: VUS (8); LB (1)	SNV	Missense
c.5487G>T	p.Leu1829Phe	CIP: VUS (6); LB (1)	SNV	Missense
c.5499T>C	p.Asn1833=	LB	SNV	synonymous
c.5740A>G	p.Ser1914Gly	NR	SNV	Missense
c.5753A>C	p.His1918Pro	NR	SNV	Missense
c.5975C>T	p.Ser1992Leu	CIP: VUS (2); LB (3)	SNV	Missense
c.6080G>A	p.Arg2027Lys	CIP: VUS (7); LB (1)	SNV	Missense
c.6092del	p.Thr2031fs	P	Deletion	Frameshift
c.6131G>C	p.Gly2044Ala	CIP: VUS (2); B (2); LB (7)	SNV	Missense
c.6290C>T	p.Thr2097Met	B	SNV	Missense
c.631+7A>G		CIP: VUS (3); LB (1)	SNV	intron variant
c.632-3_632-2del		VUS	Deletion	splice acceptor
c.6408_6411dup	p.Val2138fs	P	Duplication	Frameshift
c.6486_6489del	p.Lys2162fs	P	Deletion	Frameshift
c.6491_6494del	p.Gln2164fs	P	Deletion	Frameshift

c.6498A>G	p.Val2166=	LB	SNV	synonymous
c.6521T>C	p.Val2174Ala	CIP: VUS (3); LB (1)	SNV	Missense
c.67+1G>A		P	SNV	splice donor
c.6767G>A	p.Cys2256Tyr	CIP: VUS (2); LB (1)	SNV	Missense
c.68-7T>A		B	SNV	intron variant
c.6953G>A	p.Arg2318Gln	B	SNV	Missense
c.7048A>C	p.Thr2350Pro	VUS	SNV	Missense
c.7051G>C	p.Ala2351Pro	VUS	SNV	Missense
c.7073C>G	p.Ser2358Cys	VUS	SNV	Missense
c.7205C>G	p.Pro2402Arg	VUS	SNV	Missense
c.7397T>C	p.Val2466Ala	B	SNV	Missense
c.7435+10G>A		CIP: VUS (2); B (2); LB (3)	SNV	Frameshift
c.7481G>A	p.Arg2494Gln	CIP: VUS (3); LB (4)	SNV	Missense
c.7544C>T	p.Thr2515Ile	B	SNV	Missense
c.7618C>T	p.Leu2540=	NR	SNV	synonymous
c.771_775del	p.Asn257fs	P	Deletion	Frameshift

c.7877G>A	p.Trp2626Ter	P	SNV	nonsense
c.794-17G>A		LB	SNV	intron variant
c.8023A>G	p.Ile2675Val	P	SNV	Missense
c.8092G>A	p.Ala2698Thr	CIP: VUS (6); LB (7)	SNV	Missense
c.8261A>G	p.His2754Arg	VUS	SNV	Missense
c.8299C>T	p.Pro2767Ser	CIP: VUS (4); LB (1)	SNV	Missense
c.8324T>G	p.Met2775Arg	CIP: VUS (7); LB (2)	SNV	Missense
c.8394_8396delinsAA	p.Arg2799fs	P	Indel	Frameshift
c.8452G>A	p.Val2818Ile	CIP: VUS (7); LB (1)	SNV	Missense
c.8456A>T	p.Asp2819Val	VUS	SNV	Missense
c.8754+1G>T		P/LP	SNV	splice donor
c.8851G>A	p.Ala2951Thr	B	SNV	Missense
c.8985T>A	p.Asp2995Glu	NR	SNV	Missense
c.8995_8996del	p.Leu2999fs	LP	Deletion	Frameshift
c.9027del	p.His3010fs	P	Deletion	Frameshift
c.9097dup	p.Thr3033fs	P	Duplication	Frameshift

c.9117+3A>G		CIP:	SNV	intron variant
c.9117G>A	p.Pro3039=	P	SNV	synonymous
c.9118-1G>C		P	SNV	splice acceptor
c.9118-24G>A		NR	SNV	Noncoding
c.9242T>C	p.Val3081Ala	CIP: VUS (5); LB (6)	SNV	Missense
c.9275A>G	p.Tyr3092Cys	CIP: VUS (2); B (1); LB (9)	SNV	Missense
c.9370_9383delinsCT	p.Asn3124_Arg3128delinsLeu	VUS	Indel	inframe indel
c.9435_9443del	p.Phe3146_Ala3148del	VUS	Deletion	inframe deletion
c.9586A>G	p.Lys3196Glu	CIP: VUS (1); B (3); LB (6)	SNV	Missense
c.9670A>G	p.Ile3224Val	VUS	SNV	Missense
c.9919_9932del	p.Lys3307HisfsTer15	NR	Deletion	Frameshift
c.9976A>T	p.Lys3326Ter	B	SNV	nonsense

The transcript number is NM_000059.4(BRCA2), CIP: Conflicting interpretations of pathogenicity, NR: Not reported in ClinVar, B/LB: Benign/Likely Benign, P/LP: Pathogenic/Likely Pathogenic, VUS: Variants of uncertain significance, SNV: Single nucleotide variation.

Supporting Information Table S2. List of *BRCA2* missense VUS/CIP/NR SNV, individuals' cancer type, and non participation reason

**Case #**	**Participation**	**Nucleotide**	**Protein**	**Clinical Significance**	**Cancer Type**
42	lost to follow-up	c.10078A>G	p.Lys3360Glu	VUS	BC
56	Travel problems	c.4205A>G	p.Asn1402Ser	CIP: VUS (1), LB (1)	BC
63	lost to follow-up	c.2779A>G	p.Met927Val	CIP: VUS (6); LB (5)	BC
80	have pathogenic variation in other genes	c.5125G>T	p.Asp1709Tyr	CIP: VUS (8); LB (1)	BC
85	Not HBOC	c.6767G>A	p.Cys2256Tyr	CIP: VUS (2; LB (1	BC
88	died	c.3503T>A	p.Met1168Lys	CIP: VUS (6); LB (1)	OC
99	lost to follow-up	c.8324T>G	p.Met2775Arg	CIP: VUS (7); LB (2)	OC
103	lost to follow-up	c.7051G>C	p.Ala2351Pro	VUS	OC
107	did not want to participate	c.2240A>G	p.Glu747Gly	CIP: VUS (9); B (1); LB (1)	BC
111	male patient, not HBOC	c.8299C>T	p.Pro2767Ser	CIP: VUS (4; LB (1)	TC
**132**	**participated PC**	**c.4277C>T**	**p.Thr1426Ile**	**CIP: VUS (5); LB (2)**	**BC**
144	did not want to participate	c.9586A>G	p.Lys3196Glu	CIP: VUS (1); B (3); LB (6)	BC

150	died	c.10240A>G	p.Thr3414Ala	CIP: VUS (3); LB (5)	BC
167	have pathogenic variation in other genes	c.8261A>G	p.His2754Arg	VUS	BC
180	have pathogenic variation in other genes	c.3302A>G	p.His1101Arg	CIP: VUS (6; LB (3	BC
197	lost to follow-up	c.5740A>G	p.Ser1914Gly	NR	BC
271	have pathogenic variation in other genes	c.5125G>T	p.Asp1709Tyr	CIP	BC
294	died	c.8092G>A	p.Ala2698Thr	CIP: VUS (6); LB (7)	OC
313*	Not HBOC	c.3310A>C	p.Thr1104Pro	CIP: VUS (6); LB (1)	EC
313*	Not HBOC	c.3503T>A	p.Met1168Lys	CIP: VUS (6); LB (1)	EC
315	lost to follow-up	c.1688G>C	p.Trp563Ser	NR	CRC
319	Travel problems	c.7205C>G	p.Pro2402Arg	VUS	BC
389	Travel problems	c.6080G>A	p.Arg2027Lys	CIP: VUS (7); LB (1)	BC
**396**	**participated**	**c.3032C>G**	**p.Thr1011Arg**	**CIP: VUS (9); LB (4)**	**BC**
400	lost to follow-up	c.8452G>A	p.Val2818Ile	CIP: VUS (7); LB (1)	BC

412	lost to follow-up	c.3910A>C	p.Thr1304Pro	NR	OC
**427**	**participated**	**c.8456A>T**	**p.Asp2819Val**	**VUS**	**BC**
439	have variants in other genes	c.7481G>A	p.Arg2494Gln	CIP: VUS (8); LB (1)	-
465	lost to follow-up	c.5125G>T	p.Asp1709Tyr	CIP: VUS (8); LB (1)	BC
**466***	**participated**	**c.3310A>C**	**p.Thr1104Pro**	**CIP: VUS (6); LB (1)**	**BC**
**466***	**participated**	c.3503T>A	p.Met1168Lys	CIP: VUS (6); LB (1)	BC
504	Not HBOC	c.2467G>A	p.Ala823Thr	CIP: VUS (1); LB (1)	EC
535	lost to follow-up	c.5487G>T	p.Leu1829Phe	CIP: VUS (6); LB (1)	BC, OC
549	Not HBOC	c.8985T>A	p.Asp2995Glu	NR	GC
574	lost to follow-up	c.3318C>G	p.Ser1106Arg	CIP: VUS (5); LB (1)	BC
**666**	**participated**	**c.6080G>A**	**p.Arg2027Lys**	**CIP: VUS (7); LB (1)**	**BC**
**676**	**participated**	**c.6131G>C**	**p.Gly2044Ala**	**CIP: VUS (2); B (2); LB (7)**	**BC**
811	lost to follow-up	c.7048A>C	p.Thr2350Pro	VUS	BC
832	male patient, not HBOC	c.9242T>C	p.Val3081Ala	CIP: VUS (5); LB (6)	CRC
852	male patient	c.6521T>C	p.Val2174Ala	CIP: VUS (3); LB (1)	BC

854	have pathogenic variation in other genes	c.7073C>G	p.Ser2358Cys	VUS	BC
913	lost to follow-up	c.5975C>T	p.Ser1992Leu	CIP: VUS (2); LB (3)	BC
922*	lost to follow-up	c.4327T>C	p.Phe1443Leu	CIP: VUS (1); LB (1)	OC
922*	lost to follow-up	c.5753A>C	p.His1918Pro	NR	OC
957	lost to follow-up	c.6080G>A	p.Arg2027Lys	CIP: VUS (7); LB (1)	OC
1039	died	c.7051G>C	p.Ala2351Pro	VUS	EC
1075	have variants in other genes	c.9275A>G	p.Tyr3092Cys	CIP: VUS (2); B (1); LB (9)	-
1076*	Travel problems	c.4957A>G	p.Thr1653Ala	CIP: VUS (7); LB (2)	BC, EC
1076*	Travel problems	c.9670A>G	p.Ile3224Val	VUS	BC, EC

Transcript number is NM_000059.4 (BRCA2), *Carrying more than one BRCA2 variant, CIP: Conflicting interpretations of pathogenicity, NR: Not reported in ClinVar, B/LB: Benign/Likely Benign, P/LP: Pathogenic/Likely Pathogenic, VUS: Variants of uncertain significance, TC: Testicular Cancer, BC: Breast Cancer, OC: Ovarian Cancer, EC: Endometrial Cancer, CRC: Colorectal Cancer, GC: Gastric cancer,

PC: Pathogenic carrier

Supporting Information Table S3. Cancer and tumor type of participants, first diagnosis age, and family history

Variable	PC	T1011R	T1104P M1168K	R2027K	G2044A	D2819V
**Cancer Type**	BC	BC	BC	BC	BC	BC
**Tumor Type**	Multifocal triple (-) invasive BC	IDC (NST)	Inflammatory BC	Residual Mucinous Carcinoma	IDC	IDC
**First Diagnosis Age**	31	35	48	38	32	54
**Family History**	Mother: BC (Dx: 62)	Mother: BC (Dx: 35)	Mother: OC (Dx: 46),	Aunt: CRC (Dx: 60)	Sister 1& 2: fibrocystic breast	Mother: BC (Dx: 54)
	Aunt: Uterus Ca. and Breast Ca. (Dx: 50),	Grandmother: Brain tumor (Dx: n.a.)	Father: CRC (Dx: 65)	Grandfather: Lung Ca. (Dx: n.a.)	Cousin: BC (Dx: 44)	
	Grandmother: OC (Dx: 60)	Cousin: BC (Dx: n.a.)	Aunt: EC (Dx: n.a.)			

BC: Breast Cancer, OC: Ovarian Cancer, EC: Endometrial Cancer, CRC: Colorectal Cancer, IDC: Invasive Ductal Carcinoma, NST: no special type, PC: Pathogenic carrier.

Supporting Information Table S4. Participants' questionnaire

VARIABLE	Non-Carrier (NC1)	Non-Carrier (NC2)	Pathogenic Carrier (PC)	T1011R	T1104P M1168K	R2027K	G2044A	D2819V
**Age at interview**	38	58	37	39	52	50	36	58
**Age of diagnosis**	-	-	31	35	48	38	32	54
**Age of first birth**	-	21	23	29	23	20	28	26
**Age of menstruate**	12	13	12	12	12	15	13	13
**Height (cm)**	158	162	163	156	162	158	168	164
**Weight (kg)**	58	98	82	46	77	53.5	60	51
**BMI (kg/m^2)**	24.1	37.3	30.9	18.9	29.3	21.4	21.3	19
**Smoking statues**	Yes	No	Yes	used to	used to	used to	used to	No
**Alcohol use**	Yes	No	No	No	No	No	No	No
**Activity**	Daily	Daily	Walking	Walking	Walking	Daily	Not Active	Daily
**Use of contraceptive pill**	Used 10 years	No	No	No	Used for 5 years	Used for 1 year	No	No
**Prophylactic surgery**	No	No	Yes	No	No	No	No	No
**Exogenous Estrogens**	No	No	No	No	No	No	No	No

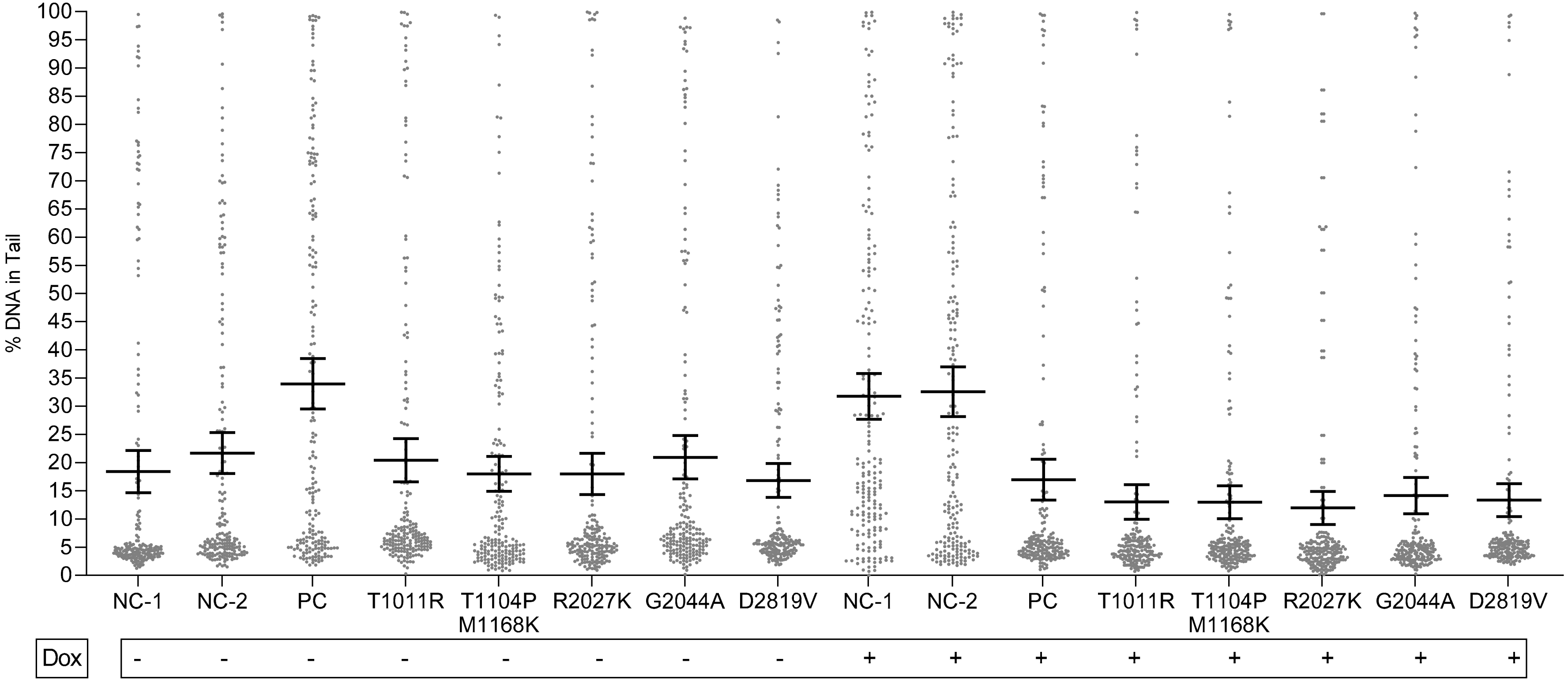

Figure S1. All subjects' percentage DNA in comet tails. Mean values with % 95 Confidence Interval. The data are representative of two independent experiments. 0.5 µM Dox-induced for 1 h. Dox: Doxorubicin, NC: Non-carrier, PC: Pathogenic variant carrier subject; BRCA1 frameshift (K711fs) deletion and a BRCA2 (T1426I) VUS, VUS carriers: VUS-C1 (T1011R), VUS-C2 (T1104P) and (M1168K), VUS-C3 (R2027K), VUS-C4 (G2044A), VUS-C5 (D2819V).

Supporting Information Table Legends

Table S1. A list of all *BRCA2* variants in the cohort

Table S2. List of *BRCA2* missense VUS/CIP/NR SNV, individuals' cancer type, and non participation reason

Table S3. Cancer and tumor type of participants, first diagnosis age, and family history

Table S4. Participants questionnaire

Supporting Information Figure Legend

Figure S1. All subjects' percentage DNA in comet tails.

Supporting Information References

11. Richards, S.; Aziz, N.; Bale, S.; Bick, D.; Das, S.; Gastier-Foster, J.; Grody, W. W.; Hegde, M.; Lyon, E.; Spector, E.; et al. Standards and guidelines for the interpretation of sequence variants: a joint consensus recommendation of the American College of Medical Genetics and Genomics and the Association for Molecular Pathology. *Genet Med* **2015**, *17*, 405-424, doi:10.1038/gim.2015.30.

13. Landrum, M. J.; Lee, J. M.; Benson, M.; Brown, G. R.; Chao, C.; Chitipiralla, S.; Gu, B.; Hart, J.; Hoffman, D.; Jang, W.; et al. ClinVar: improving access to variant interpretations and supporting evidence. *Nucleic Acids Res* **2018**, *46*, D1062-d1067, doi:10.1093/nar/gkx1153.

18. Akcay, I. M.; Celik, E.; Agaoglu, N. B.; Alkurt, G.; Kizilboga Akgun, T.; Yildiz, J.; Enc, F.; Kir, G.; Canbek, S.; Kilic, A.; et al. Germline pathogenic variant spectrum in 25 cancer susceptibility genes in Turkish breast and colorectal cancer patients and elderly controls. *Int J Cancer* **2021**, *148*, 285-295, doi:10.1002/ijc.33199.

19. Ogulur, I.; Ertuzun, T.; Kocamis, B.; Kendir Demirkol, Y.; Uyar, E.; Kiykim, A.; Baser, D.; Yesil, G.; Akturk, H.; Somer, A.; et al. Parents of ataxia-telangiectasia patients display a distinct cellular immune phenotype mimicking ATM-mutated patients. *Pediatr Allergy Immunol* **2021**, *32*, 349-357, doi:10.1111/pai.13387.

21. Karczewski, K. J.; Francioli, L. C.; Tiao, G.; Cummings, B. B.; Alföldi, J.; Wang, Q.; Collins, R. L.; Laricchia, K. M.; Ganna, A.; Birnbaum, D. P.; et al. The mutational constraint spectrum quantified from variation in 141,456 humans. *Nature* **2020**, *581*, 434-443, doi:10.1038/s41586-020-2308-7.

43. Daly, M. B.; Pal, T.; Maxwell, K. N.; Churpek, J.; Kohlmann, W.; AlHilli, Z.; Arun, B.; Buys, S. S.; Cheng, H.; Domchek, S. M.; et al. NCCN Guidelines® Insights: Genetic/Familial High-Risk Assessment: Breast, Ovarian, and Pancreatic, Version 2.2024. *J Natl Compr Canc Netw* **2023**, *21*, 1000-1010, doi:10.6004/jnccn.2023.0051.

44. Weiss, J. M.; Gupta, S.; Burke, C. A.; Axell, L.; Chen, L. M.; Chung, D. C.; Clayback, K. M.; Dallas, S.; Felder, S.; Gbolahan, O.; et al. NCCN Guidelines® Insights: Genetic/Familial High-Risk Assessment: Colorectal, Version 1.2021. *J Natl Compr Canc Netw* **2021**, *19*, 1122-1132, doi:10.1164/jnccn.2021.0048.

45. Ng, P. C.; Henikoff, S. SIFT: Predicting amino acid changes that affect protein function. *Nucleic Acids Res* **2003**, *31*, 3812-3814, doi:10.1093/nar/gkg509.

46. Adzhubei, I.; Jordan, D. M.; Sunyaev, S. R. Predicting functional effect of human missense mutations using PolyPhen-2. *Curr Protoc Hum Genet* **2013**, *Chapter 7*, Unit7.20, doi:10.1002/0471142905.hg0720s76.

47. Li, C.; Zhi, D.; Wang, K.; Liu, X. MetaRNN: differentiating rare pathogenic and rare benign missense SNVs and InDels using deep learning. *Genome Med* **2022**, *14*, 115, doi:10.1186/s13073-022-01120-z.

48. Liu, X.; Jian, X.; Boerwinkle, E. dbNSFP: a lightweight database of human nonsynonymous SNPs and their functional predictions. *Hum Mutat* **2011**, *32*, 894-899, doi:10.1002/humu.21517.

49. Liu, X.; Li, C.; Mou, C.; Dong, Y.; Tu, Y. dbNSFP v4: a comprehensive database of transcript-specific functional predictions and annotations for human nonsynonymous and splice-site SNVs. *Genome Med* **2020**, *12*, 103, doi:10.1186/s13073-020-00803-9.

50. Kopanos, C.; Tsiolkas, V.; Kouris, A.; Chapple, C. E.; Albarca Aguilera, M.; Meyer, R.; Massouras, A. VarSome: the human genomic variant search engine. *Bioinformatics* **2018**, *35*, 1978-1980, doi:10.1093/bioinformatics/bty897.

51. Luppino, F.; Adzhubei, I. A.; Cassa, C. A.; Toth-Petroczy, A. DeMAG predicts the effects of variants in clinically actionable genes by integrating structural and evolutionary epistatic features. *Nat Commun* **2023**, *14*, 2230, doi:10.1038/s41467-023-37661-z.

52. Lovell, D. P.; Omori, T. Statistical issues in the use of the comet assay. *Mutagenesis* **2008**, *23*, 171-182, doi:10.1093/mutage/gen015.
